# Seroprevalence of Brucellosis, Knowledge, and Risky Practices in Dairy Cattle Owners and Workers in Maekel and Debub Regions, Eritrea

**DOI:** 10.4269/ajtmh.23-0476

**Published:** 2024-06-18

**Authors:** Ghebremeskel Habteyohannes Efrem, Bereket Mihreteab, Michael K. Ghebremariam, Berhe Tesfai, Siobhan M. Mor, Gezahegne Mamo

**Affiliations:** ^1^ National Animal and Plant Health Laboratory, Ministry of Agriculture, Asmara, Eritrea;; ^2^ Department of Veterinary Microbiology, Immunology and Public Health, College of Veterinary Medicine and Agriculture, Addis Ababa University, Ethiopia;; ^3^ Pathology Laboratory, National Animal and Plant Health Laboratory, Ministry of Agriculture, Asmara, Eritrea;; ^4^ School of Biodiversity, One Health and Veterinary Medicine, University of Glasgow, Glasgow, United Kingdom;; ^5^ Massawa Hospital, Northern Red Sea Region Ministry of Health, Massawa, Eritrea;; ^6^ Institute of Infection, Veterinary and Ecological Sciences, University of Liverpool, Leahurst, United Kingdom;; ^7^ International Livestock Research Institution, Addis Ababa, Ethiopia

## Abstract

Brucellosis is a zoonotic disease with worldwide distribution. In Eritrea, the status of the disease in occupationally exposed dairy farmers is unknown. The objective of this study was to determine the seroprevalence of brucellosis, level of knowledge, and risky practices of dairy cattle owners/workers in Maekel and Debub regions, Eritrea. A cross-sectional study was conducted between August 2021 and February 2022. A total of 416 dairy cattle owners and workers underwent blood collection and interview using a standardized questionnaire. Blood samples were tested using Rose Bengal Plate Test, and positive samples were confirmed using competitive ELISA. Variation in knowledge scores by sociodemographic factors and practices were explored statistically. The apparent and true seroprevalence was 1.2% (95% CI: 0.05–2.8%) and 1.4% (95% CI: 0.6–3.4%), respectively. Apparent seroprevalence was similar in Maekel (1.1%) and Debub (1.2%) regions. Nearly half of the participants (49.5%) had never heard of brucellosis before. Overall, brucellosis knowledge score was low (mean score: 6.53/20). Knowledge score was higher in participants from Maekel region (*P* <0.001), older participants (*P =* 0.035), those with higher educational attainment (*P =* 0.001), and those with more years of experience working in dairy farming (*P =* 0.001). Knowledge score was lower in farm workers compared with family members (*P =* 0.016). No significant differences in knowledge score existed between participants who engaged in or did not engage in potential risky practices. In summary, the prevalence of brucellosis in dairy cattle owners/workers in Maekel and Debub regions, Eritrea, was low. Participants demonstrated limited knowledge of brucellosis and engaged in risky practices.

## INTRODUCTION

Brucellosis is a zoonotic disease that causes significant losses in livestock productivity as well as negative impacts on public health worldwide.^1^ The disease is caused by various species of the genus *Brucella* spp.^2^ with *B. melitensis*, *B. abortus*, and *B. suis* considered the most important species in terms of human health.^3^ Globally, approximately half a million new cases of human brucellosis are recorded each year.^4^ Humans acquire infection through consumption of unpasteurized dairy products and uncooked meat or through direct contact with infected animals, placentas, or aborted fetuses.^5^

Clinically, human brucellosis is characterized by fever, fatigue, sweating, joint pain, headache, loss of appetite, muscular pain, lumbar pain, weight loss, and arthritis.^5^^,^^6^ The disease is insidious and can be severely debilitating, with acute febrile episodes frequently misdiagnosed as malaria.^1^ Treatment of brucellosis involves prolonged therapy with two or more antimicrobial drugs and delay in treatment can lead to long-term complications. Consequently, brucellosis imposes a significant economic burden on society through loss of workdays due to malaise and hospitalization of infected persons, as well as loss of income and healthcare costs.^7^ Dairy farmers, pastoralists, abattoir workers, animal health personnel, laboratory personnel, and other people involved in the livestock value chain are considered the highest occupational risk groups.^8^ Adequate knowledge of brucellosis is of significant importance among high-risk groups because knowledge empowers people to take protective measures at work and actively participate in disease control programs, thus greatly assisting in brucellosis control strategies.^9^

Limited studies have been conducted on brucellosis in humans in Eritrea to date. Early this century (2002), Omer et al. investigated the disease in a small number of occupational-exposed groups including dairy farmers (*n =* 130), veterinary personnel (*n =* 21), and pastoralists (*n =* 105) with reported seropositivity of 7.1%, 4.6%, and 3.0% in each group, respectively.^10^ More recently, in 2022, Efrem et al. reported 2.4% seroprevalence in livestock owners in Northern Red Sea region (*n =* 637).^11^ Fragmented clinical reports, laboratory diagnostic results, and serological tests in livestock owners also indicate the presence of brucellosis in humans in different areas of the county.^12^^,^^13^ However, there is lack of data on the level of knowledge and practices in different farming systems, including in the dairy-cattle-rearing community.

The objective of this study was therefore to determine the seroprevalence of brucellosis and the level of knowledge and risky practices in dairy cattle workers in Maekel and Debub regions, Eritrea. These regions constitute the most densely populated regions in terms of human and dairy cattle populations in Eritrea.

## MATERIALS AND METHODS

### Study design and area.

This cross-sectional, community-based study was conducted in dairy cattle owners and workers in Maekel and Debub regions of Eritrea between August 2021 and February 2022. Eritrea is located in the Horn of Africa, between latitudes 12°42′N and 18°2′N and longitudes 36°30′E to 43°20′E. The country is divided into six administrative regions: Anseba, Debub, Gash-Barka, Maekel, Southern Red Sea, and Northern Red Sea. Debub region is located in the southern part of the country. It is divided into 12 subregions, 217 administrative areas, and 886 villages. It comprises the second highest population of all regions. Maekel region is in the central part of the country and divided into four subregions, 59 administrative areas, and 89 villages. It is the smallest in size but has the third largest population of all regions in Eritrea.^14^^,^^15^

### Sampling design and sample size.

Dairy cattle owners and workers from dairy farms managed under intensive and semi-intensive production systems in Maekel and Debub regions were the target of this study. The study was conducted simultaneously with another study that focused on prevalence of brucellosis and risk factors in dairy cattle on the same farms.^16^ The criteria for selecting the farms is described in detail in that article. Briefly, lists of dairy farms were obtained from the respective regional Ministry of Agriculture Veterinary Offices. From these, farms with more than four animals in the study areas were selected to participate, proportional to the number of farms in each region (i.e., 87 farms in Maekel; 127 farms in Debub). The mean and median herd size of the sampled farms was 15.1 and nine head of cattle, respectively (minimum five, maximum 158).

Between one and four dairy cattle owners/workers from each farm volunteered for blood and data collection, for a total of 416 human participants from 214 farms. With this sample size, using EpiTools (https://epitools.ausvet.com.au/prevalencess), we calculated that we could estimate the true seroprevalence in humans with approximately 5% precision and 95% confidence. This assumed a worst-case scenario of 30% prevalence (based on 28.4% seropositivity in clinically suspected cases^17^), and combined sensitivity and specificity for Rose Bengal plate test (RBPT) and competitive ELISA (c-ELISA) of 83% and 100%, respectively (based on data reported by Godfroid et al.^18^ and combined using the formula by Thrusfield^19^). We elected to use a high (30%) expected prevalence for these calculations because previous estimates were 20 years old,^10^ and it was assumed that prevalence would have increased in the absence of disease control efforts.

### Data and sample collection.

A questionnaire was designed to assess knowledge and risky practices related to brucellosis acquisition in humans (see supplemental material). Participants were asked if they had heard about brucellosis before, as well as sources of information, routes of transmission from animals to humans, clinical signs in infected animals and humans, and availability of treatment or vaccine. Participants were also asked if they consume milk and milk products, eat raw meat, and assist animals during parturition without protective equipment. The contents of the questionnaire were prepared using simple and easily understandable close-ended questions. The questionnaire was translated into the local language (Tigrinya) and pretested in 10 households before commencement of data collection, with adjustments made to improve clarity.

The authorities in the respective study region gave permission for the research to be conducted in the study areas. During the informed consent process, participants were advised that they could withdraw at any stage of the research and that all the information provided including any identifiable information would be kept confidential. Seven to ten ml of blood was collected from each participant from the median cubital vein using a plain vacutainer tube and double ended needle. Each blood sample was labeled using a unique code to maintain confidentiality. The sample tubes were set tilted on a table for about 12–18 hours at a room temperature and protected from direct sunlight to allow separation of the serum. Subsequently, separated serum was decanted into another tube pre-labeled with the same unique code. Serum samples were then transported in an ice box to the National Animal and Plant Health Laboratory (NAPHL), Ministry of Agriculture, Asmara for laboratory analysis.

### Laboratory investigation.

Sera was screened for brucellosis antibody using the RBPT according to the kit manufacturer’s instructions (product code RAA0060; Animal Health and Veterinary Laboratory Agency, Surry, United Kingdom). Negative and positive controls for *B. abortus* and *B. melitensis* were included at the beginning of each testing session. All RBPT positive samples were subjected to confirmation using c-ELISA following procedures described by the kit manufacturer (INgezim *Brucella* Compac 2.0; Ingenasa, Madrid, Spain). Optical density (OD) of each well was read at 450-nm using a plate-reader machine. The result was deemed acceptable if the OD in negative controls wells (NC) was >1 and OD in positive controls wells (PC) was <0.35. The percentage of inhibition (PI) for each sample was calculated as follows: PI = 100 × [1 – (OD sample/OD negative control)]. Samples with PI ≥40% were considered positive for *Brucella* antibody, and samples were deemed negative if PI <40%.

## STATISTICAL ANALYSES

Questionnaire data and laboratory results were coded and entered into Microsoft Excel and imported into SPSS version 23 for analysis. Estimated true prevalence using imperfect tests was calculated in Epitools (https://epitools.ausvet.com.au/trueprevalence) using the same parameters above. Sociodemographic background, knowledge, and self-reported practices were summarized using descriptive statistics (frequency and percentages). Knowledge score was calculated by allocating marks to each question. Participants who had never heard of brucellosis were given a score of zero. Participants who had heard of brucellosis were asked to answer an additional 12 questions consisting of a total of 20 marks. The normality distribution of knowledge score data was checked graphically with a histogram and using Kolmogorov–Smirnov tests. Because the data were not normally distributed, nonparametric tests (Mann–Whitney *U* test and Kruskal–Wallis tests) were used to explore differences in median knowledge score by demographic characteristics and engagement in risky practices. Associations between seropositivity and knowledge score and between seropositivity and risky practices were also examined using Mann–Whitney and chi-square tests, respectively. All associations were found to be nonsignificant, likely because of the low number of seropositives detected in the study (*n* = 5) (see Supplemental Materials).

## RESULTS

### Participant characteristics and seroprevalence.

Table 1 shows the characteristics of participants, by brucellosis serostatus. Of 416 participants, 8 (1.9%) were seropositive for brucellosis by RBPT, of whom five were confirmed by c-ELISA. Thus, the apparent and true individual-level prevalence was 1.2% (95% CI: 0.05–2.8%) and 1.4% (95% CI: 0.6–3.4%), respectively. All individuals came from different farms; apparent farm-level prevalence was five of 214 (2.3%). Of 416 participants tested across both regions, two of 175 (1.1%) from Maekel were seropositive and three of 241 (1.2%) from Debub were seropositive.

**Table 1:** Demographic characteristics and brucellosis serostatus of dairy farm owners and workers in Maekal and Debub regions, Eritrea (*N* = 416)

Variable	Total Samples, *n* (%)	c-ELISA Positive, *n* (%)
Region		
Maekel	175 (42.1)	2 (1.1)
Debub	241 (57.9)	3 (1.2)
Age in years		
18–30	147 (35.3)	3 (2.0)
31–50	154 (37)	2 (1.3)
>50	115 (27.6)	0 (0.0)
Gender		
Male	310 (74.5)	4 (1.3)
Female	106 (25.5)	1 (0.9)
Educational level		
Illiterate (0)	40 (9.6)	0 (0.0)
Primary (1–5)	89 (21.4)	1 (1.1)
Junior and secondary (6–12)	250 (60.1)	3 (1.2)
Higher education (>12)	37 (8.9)	1 (2.7)
Relation of respondent to household		
Family member	268 (64.4)	2 (0.7)
Farm worker	148 (35.6)	3 (2.0)
Years working in dairy farming		
≤10	231 (55.8)	5 (2.2)
11–30	164 (39.6)	0 (0.0)
>30	19 (4.6)	0 (0.0)
No. of household members		
1–5	154 (37.0)	2 (1.3)
6–10	196 (47.1)	2 (1.0)
11–20	66 (15.9)	1 (1.5)
Total	416	5 (1.2)

### Brucellosis knowledge of participants.

Brucellosis knowledge of participants is summarized in the supplemental material. Nearly half of the participants (206/416; 49.5%) had never heard of brucellosis before. Of 210 participants (50.5%) who had heard of brucellosis, respondents stated that they had heard of brucellosis from television (123/210; 59%), friends/relatives (65/210; 31%), and radio (34/210; 16%); through community awareness-raising programs (20/210; 9.5%); and/or from newspapers (2/210; 1%). The great majority (196/210; 94%) of these participants knew that animals can be infected with brucellosis. Animal species that were identified as being infected with brucellosis were cattle (192/210; 91%), sheep/goats (142/210; 67%), and camels (47/210; 22%). Respondents correctly identified several clinical signs of brucellosis in animals including abortion (113/210; 54%), repeat breeding (25/210; 12%), loss of milk production (21/210; 10%), infertility (10/210; 5%), retained placenta (10/210; 5%), and epididymitis (4/210; 2%). However, 41% (86/210) of participants did not know any clinical sign of brucellosis in animals. Nearly 85% (178/210) of respondents indicated that brucellosis can be transmitted from animals to humans by eating raw meat or drinking raw milk. More than half (110/210; 52%) answered that brucellosis can be transmitted to humans through direct contact with contaminated fluids and tissues from animals with brucellosis. Brucellosis symptoms and clinical signs in humans were recognized by some participants and included joint and back pain (74/210; 35%), fever (46/210; 22%), weakness (36/210; 17%), and headache (20/210; 10%). However, 49% (103/210) did not know any symptoms of brucellosis in humans. Most (181/210; 86%) respondents answered that brucellosis can be medically treated in humans, and 70% (146/210) knew that a vaccine against brucellosis in animals is available.

### Self-reported practices.

Self-reported practices of participants are summarized in the supplemental material. Of 416 participants, 96% (*n* = 400) reported that they consume boiled/pasteurized milk. Most of them (290/416; 70%) explained that yoghurt is commonly made from raw milk and is further processed to butter and buttermilk for home consumption and sale. Participants indicated that cheese was not processed at home; if any exists, it was produced in dairy processing plants. Only a small proportion (31/416; 7.5%) had the habit of eating raw meat, and it was stated to happen only rarely on holidays. The majority (311/416; 75%) of respondents stated that they assisted animals during parturition, and most (189/311; 61%) stated that they did not wear protective gear.

### Comparison of knowledge score by demographic characteristics and engagement in risky practices.

Overall brucellosis knowledge score of participants was low (mean score of 6.53/20; median of 1, interquartile range [IQR]: 0–12.5). Significant differences existed in median knowledge score by demographic characteristics (Table 2). Knowledge score was higher in participants from Maekel region (*P* <0.001), older participants (*P* = 0.035), those with higher educational attainment (*P* = 0.001), and those with more years of experience working in dairy farming (*P* = 0.001). In contrast, knowledge score was lower in farm workers (versus family members; *P* = 0.016). No significant differences in knowledge score existed between participants who engaged in potential risky practices including drinking yoghurt produced from raw milk (*P* = 0.874), eating raw meat (*P* = 0.197), and assisting calving animals without wearing protective gloves (*P* = 0.100), compared with those who did not engage in such practices (not shown).

**Table 2:** Median brucellosis knowledge score of dairy workers in Maekal and Debub regions, Eritrea, by demographic characteristics (*N* = 416)

Variable	Median Value of Knowledge Score	*P*-Value
Region		
Maekel	11	<0.001
Debub	0	
Age		
18–30	0	0.035
31–50	3.5	
>50	10	
Gender		
Male	0	0.767
Female	5.5	
Educational level		
Illiterate (0)	0	0.001
Primary (1–5)	0	
Junior and secondary (6–2)	2.5	
Higher education (>12)	11	
Relation of respondent to household		
Family member	7.5	0.016
Farm worker	0	
Years in working in dairy farming		
≤10	0	0.001
11–30	9	
>30	10	
No. of household members		
1–5	0	0.922
6–10	5	
11–20	0	

*P*-values comparing knowledge score between categories were derived using Mann–Whitney *U* test for two samples and Kruskal–Wallis test for three or more samples.

## DISCUSSION

This study is one of few to assess the seroprevalence of brucellosis and associated knowledge and practices related to brucellosis in dairy farmers and workers in Eritrea. The overall apparent seroprevalence of brucellosis in dairy farmers in this study (1.2%) was lower than reported 20 years previously in a study of dairy farm workers around the capital city, Asmara (7%).^10^ It was also slightly lower than reported more recently in livestock owners in Northern Red Sea region (2.4%).^11^ Interestingly, the current study in humans was simultaneously carried out with a seroprevalence survey of dairy cattle on the same farms where the prevalence was found to be low; 1.2% and 1.1% in cattle in Maekal and Debub regions, respectively.^16^ Simultaneous sampling of humans and animals is uncommon in brucellosis research and is a strength of this study. The findings agree with the common understanding that the epidemiology of brucellosis among humans largely reflects the epidemiology in animals.^20^

Nearly half of the dairy cattle farmers in the present study had never heard of brucellosis before. Comparable observations have been reported in South Africa where more than 40% of farmers had not heard about brucellosis.^21^ In contrast, in neighboring Ethiopia, more than 90% of dairy farm workers had never heard about brucellosis.^22^ Similar findings have been reported in India and Tajikistan.^23^^,^^24^ Shortage of health facilities, inadequate veterinary services, shortage of personal protective gear, lack of training on the rearing and handling of animals and lack of health education programs have previously been reported as the major contributors to the low level of awareness/knowledge among dairy farmers.^25^

Of the farmers who had heard about brucellosis, most answered that they acquired the information through mass media (television and radio). Most likely this high proportion was linked with the brucellosis-related information broadcasted on weekly basis for several months in the years 2020–2021 (Eri-TV-programs 2020/21). Nowadays, Eri-TV and radio are the most accessible and commonly consumed mass media by almost all ages and sexes in the country. Hence, it is important that the concerned authorities and experts continue to use the mass media to convey and educate the community about this disease. In particular, most farmers in this study had poor knowledge of clinical signs of brucellosis in animals and humans. This is an important finding and suggests that farmers may not recognize brucellosis when it occurs in their herd, nor may they attribute signs they experience to the disease. This knowledge gap could be targeted in future mass media campaigns.

Drinking raw milk is an important risk factor for acquiring brucellosis infection in humans. Presence and viability of *Brucella* species in dairy products is influenced by various intrinsic and extrinsic factors such as temperature, moisture content, pH, storage condition, age, and type of dairy product.^26^ It is generally recommended that all milk for human consumption should be boiled or pasteurized at high temperatures to kill *Brucella* spp. If pasteurization facilities are not available, the milk should be heated to a minimum temperature of 80 to 85°C and held at that level for at least 30 minutes.^1^ In this study, most participants stated that they drink only boiled or pasteurized milk. This is a good preventive measure and likely contributes to the low observed seroprevalence in the study areas. This contrasts with a study of livestock owners in the Northern Red Sea region of Eritrea, where most respondents (77.8%) reported that they never boiled milk before drinking or selling.^11^ These regional differences may be important for designing appropriately targeted messaging campaigns. Notably, participants in the current study did report that yoghurt was commonly made from raw milk, and it was further processed to butter and buttermilk for home consumption and sale. This is a risky practice and custom, highlighting the need to target people engaged in downstream milk processing in education campaigns in addition to primary producers.

Most participants in this study assisted their animals during parturition and did not wear protective gear while doing so. This finding is consistent with a previous study conducted in Eritrea^11^ and is similar to another in Egypt.^27^ The results are, however, in contrast to a study conducted in Jordan,^26^ where nearly half of the respondents indicated that they wore gloves for protection. In Eritrea, this risky practice could be partly explained by the fact that participants cannot easily find such protective equipment in their vicinity. This constraint should be recognized by concerned extension workers who are advised to explore and find solutions.

Higher knowledge scores were observed for participants from Maekel region than Debub, probably because of the relatively longer history of dairy farming in Maekel region, where more studies have been conducted. Hence dairy owners and other stakeholders involved in the dairy chain and residing in Maekel region could have been exposed to better information about brucellosis. Three demographic factors (increasing age, educational level, and number of years in dairy cattle work) showed a significant positive association with higher knowledge score. This likely reflects cumulative exposure to information and associated learning through various sources.

There are some limitations of this study. First, the study excluded farms owning fewer than five animals. Therefore, the findings cannot be generalized to small farms. Our aim was to measure seroprevalence, and as such we applied serological tests at a single point in time. We cannot comment on whether any of the seropositive participants had active brucellosis infection or whether participants who were seronegative had been exposed in the past. The small number of seropositives also prohibited detailed analysis of risk factors using multivariable analysis. Further, the kits that were used for serological screening and confirmation in humans in this study were developed for use in animals. We did not undertake a full evaluation of these tests; however, basic validation was performed using control sera before application of the tests to human samples in this study. We also used the same cutoff value as established in other studies that used a c-ELISA kit developed and validated for livestock as a confirmatory test in humans.^28^^,^^29^ Finally, potential biases could also have arisen if the questions were misinterpreted by the farmers. Every effort was taken to prevent this by pretesting the questionnaire.

## CONCLUSION

In conclusion, the prevalence of brucellosis in dairy cattle owners and workers in Maekel and Debub regions of Eritrea was low. Participants had low levels of knowledge on brucellosis and engaged in practices that posed potential risks related to the disease. Dairy cattle owners and workers, and the communities in the study areas in general, need to be educated on brucellosis and related risky practices and preventive methods. Concerned authorities and extension workers should make every effort to expand access of dairy owners and workers to protective equipment including gloves.

## Supplemental Materials

10.4269/ajtmh.23-0476 Supplemental Materials

## References

[1] Zoonotic Disease Unit , 2021. National Strategy for the Prevention and Control of Brucellosis in Humans and Animals in Kenya (2021–2040). Nairobi, Kenya: Ministry of Agriculture, Livestock, Fisheries and Cooperatives, Ministry of Health.

[2] Adone R, 2013. Epidemiosurveillance of brucellosis. Rev Sci Tech 32: 199–205. 23837377 10.20506/rst.32.1.2202

[3] Young EJ, 1995. An overview of human brucellosis. Clin Infect Dis 21: 283–290. 8562733 10.1093/clinids/21.2.283

[4] Godfroid J, 2017. Brucellosis in livestock and wildlife: Zoonotic diseases without pandemic potential in need of innovative One Health approaches. Arch Public Health 75: 34. 28904791 10.1186/s13690-017-0207-7 PMC5592711

[5] Dean AS Crump L Greter H Hattendorf J Schelling E Zinsstag J, 2012. Clinical manifestations of human brucellosis: A systematic review and meta-analysis. PLoS Negl Trop Dis 6: e1929. 23236528 10.1371/journal.pntd.0001929 PMC3516581

[6] Franco MP Mulder M Gilman RH Smits HL, 2007. Human brucellosis. Lancet Infect Dis 7: 775–786. 18045560 10.1016/S1473-3099(07)70286-4

[7] Franc KA Krecek RC Hasler BN Arenas-Gamboa AM, 2018. Brucellosis remains a neglected disease in the developing world: A call for interdisciplinary action. BMC Public Health 18: 125–132. 29325516 10.1186/s12889-017-5016-y PMC5765637

[8] Al-Shamahy HA Whitty CJ Akbarmehr J, 2011. The prevalence of *Brucella abortus* and *Brucella melitensis* in local cheese produced in Sarab city, Iran and its public health implication. Afr J Microbiol Res 5: 1500–1503.

[9] Zhang N Zhou H Huang DS Guan P, 2019. Brucellosis awareness and knowledge in communities worldwide: A systematic review and meta-analysis of 79 observational studies. PLoS Negl Trop Dis 13: e0007366. 31048848 10.1371/journal.pntd.0007366 PMC6497230

[10] Omer MK Assefaw T Skjerve E Tekleghiorghis T Woldehiwet Z, 2002. Prevalence of antibodies to *Brucella* spp. and risk factors related to high-risk occupational groups in Eritrea. Epidemiol Infect 129: 85–91. 12211600 10.1017/s0950268802007215 PMC2869878

[11] Efrem GH , 2022. Prevalence of brucellosis in livestock owners in Northern Red Sea Region, Eritrea: Community knowledge, attitude and practice. Arch Infect Dis Ther 6: 215–226.

[12] Weldegiorgis BM Mesfin AB Beyene NL Afewerki YP Abdelkadr MW Tsegai TO Nuwayu FN Araia ZZ, 2021. Investigation of brucellosis using molecular and serological tools in selected sites of Maekel region, Eritrea. Eur J Biol Biotechnol 2: 92–96.

[13] National Animal and Plant Health Laboratory , 2021. *Annual Report 2021*. Asmara, Eritrea: Ministry of Agriculture.

[14] Bahta YT Haile BO, 2012. Determinants of poverty of Zoba Maekel of Eritrea: A household level analysis. Int J Food Agric Econ 1: 73–84.

[15] Ministry of Agriculture , 2019. Annual Report, 2019. Asmara, Eritrea: Agricultural Extension Department, Animal Health Division.

[16] Efrem GH Mihreteab B Ghebremariam MK Okbamichael T Ghebresilasie Y Mor SM Mamo G, 2023. Prevalence of brucellosis and associated risk factors in dairy cattle in Maekel and Debub regions, Eritrea. Front Vet Sci 10: 1177572. 37396997 10.3389/fvets.2023.1177572 PMC10311105

[17] National Animal and Plant Health Laboratory , 2017. Annual Report 2017. Asmara, Eritrea: Ministry of Agriculture.

[18] Godfroid J Nielsen K Saegerman C, 2010. Diagnosis of brucellosis in livestock and wildlife. Croat Med J 51: 296–305. 20718082 10.3325/cmj.2010.51.296 PMC2931434

[19] Thrusfield MV, 2005. Veterinary Epidemiology. 3rd ed. Oxford, United Kingdom: Blackwell Science Ltd., 168–205.

[20] Glynn MK Lynn TV, 2008. Brucellosis. J Am Vet Med Assoc 233: 900–908. 18795849 10.2460/javma.233.6.900

[21] Cloete A Gerstenberg C Mayet N Tempia S, 2019. Brucellosis knowledge, attitudes and practices of a South African communal cattle keeper group’. Onderstepoort J Vet Res 86: a1671. 10.4102/ojvr.v86i1.1671 PMC6407466 30843408

[22] Edao BM Hailegebreal G Berg S Zewude A Zeleke Y Sori T Almaw G Whatmore AM Ameni G James LNW, 2018. Brucellosis in the Addis Ababa dairy cattle: The myths and the realities. BMC Vet Res 2018: 396. 10.1186/s12917-018-1709-4 PMC6293529 30547772

[23] Deka RP Magnusson U Grace D Shome R Lindahl JF, 2020. Knowledge and practices of dairy farmers relating to brucellosis in urban, periurban and rural areas of Assam and Bihar, India. Infect Ecol Epidemiol 10: 1769531. 33224446 10.1080/20008686.2020.1769531 PMC7655058

[24] Lindahl E Sattorov N Boqvist S Magnusson U, 2015. A study of knowledge, attitudes and practices relating to brucellosis among small-scale dairy farmers in an urban and peri-urban area of Tajikistan. PLoS One 10: e0117318. 25668783 10.1371/journal.pone.0117318 PMC4323107

[25] Munyeme M Muma JB Munang’andu HM Kankya C Skjerve E Tryland M, 2010. Cattle owners’ awareness of bovine tuberculosis in high and low prevalence settings of the wildlife-livestock interface areas in Zambia. BMC Vet Res 6: 21. 20406464 10.1186/1746-6148-6-21 PMC2874791

[26] Musallam II Abo-Shehada MN Guitian J, 2015. Knowledge, attitudes, and practices associated with brucellosis in livestock owners in Jordan. Am J Trop Med Hyg 93: 1148–1155. 26438029 10.4269/ajtmh.15-0294 PMC4674226

[27] Holt HR Eltholth MM Hegazy YM El-Tras WF Tayel AA Guitian J, 2011. *Brucella* spp. infection in large ruminants in an endemic area of Egypt: Cross-sectional study investigating seroprevalence, risk factors and livestock owner’s knowledge, attitudes and practices (KAPs). BMC Public Health 11: 341. 21595871 10.1186/1471-2458-11-341 PMC3121632

[28] Shirima GM Kunda JS, 2016. Prevalence of brucellosis in the human, livestock and wildlife interface areas of Serengeti National Park, Tanzania. Onderstepoort J Vet Res 83: a1032. 27247075 10.4102/ojvr.v83i1.1032 PMC6238685

[29] John K Fitzpatrick J French N Kazwala R Kambarage D Mfinanga GS MacMillan A Cleaveland S, 2010. Quantifying risk factors for human brucellosis in rural northern Tanzania. PLoS One 5: e9968. 20376363 10.1371/journal.pone.0009968 PMC2848606

