# Supplementary material for: Seroprevalence of Brucellosis, Knowledge, and Risky Practices in Dairy Cattle Owners and Workers in Maekel and Debub Regions, Eritrea
Source: Am J Trop Med Hyg. 2024 Jun 18;111(2):300–4. doi: 10.4269/ajtmh.23-0476 (PMC11310605; doi:10.4269/ajtmh.23-0476)
Supplement: Supplemental Materials [file tpmd230476.SD1.pdf]

S1: Questionnaire to assess brucellosis knowledge and practices in dairy cattle owners/workers in Maekel and Debub regions, Eritrea

SECTION-1: Socio-demographic data of respondent

Code	Questions	Response
	Respondent's ID /Code [ ]	Supervisor:________________
	Name of the respondent:____________________<br>______________________________<br>Tel No:__________________________________	1. Zoba____________________<br>2. Sub-zoba ______________<br>3. Adm /Kebabi ____________<br>4. Village__________________
SD1	Age of the respondent, in years:	[ ]
SD2	Sex of the respondent	1. Male<br>2. Female
SD3	Relation of the person interviewed to the house hold (HH)?	1. Owner/head<br>2. Spouse<br>3. Child<br>4. Farm worker/attendant
SD4	For how long have you been worked on dairy farming? in years	[ ]
SD5	Educational Level of the respondent	1. Illiterate<br>2. Primary school<br>3. Junior and secondary school<br>4. Higher education
SD6	What is the total number of Household Members, currently	[ ]

SECTION-2: Human brucellosis risk assessment

Code	Questions	Response
HR1	Do you drink/eat milk and/or milk products?	1. Yes<br>2. No
HR2	If yes to HR-1 what type of milk or milk product?	1. Fresh /raw milk<br>2. Boiled /pasteurized milk<br>3. Products from raw milk<br>4. Products from boiled/ pasteurized milk
HR3	Do you eat meat?	1. Yes<br>2. No
HR4	If yes to HR 9, how is the meat prepared /served ?	1. Raw<br>2. Cooked
HR5	Which type of meat /organs do you have a habit of eating raw?	1. Red meat<br>2. Liver<br>3. Blood<br>4. None

HR6	During birth/calving time, do you assist your animal(s)?	1. Yes<br>2. No
HR 7	If yes to MH6, do you wear protective gears (*glove, boot, mask, overall*) ?	1. Yes<br>2. No
HR 8	How do you handle and dispose birth materials (*aborted fetus, placenta, contaminated soil*) ?	1. Wearing protective gears<br>2. Without wearing gears
HR 9	Have you ever been diagnosed with *brucellosis*?	1. Yes<br>2. No
HR 10	If yes to HR9, indicate the date/month /year	

SECTION-3: Brucellosis awareness/knowledge assessment

Code	Questions	Response
AK1	Have you ever heard of the disease Brucellosis?	1. Yes<br>2. No
AK2	Can humans be infected with Brucellosis?	1. Yes<br>2. No
AK3	Do cattle transmit Brucellosis to humans?	1. Yes<br>2. No
AK4	Do you know that any member of the family is at risk of acquiring brucellosis?	1. Yes<br>2. No
AK5	Do you know how humans can be infected from animals (routes of infection)?	1. Yes<br>2. No
AK6	Do you know Brucellosis in humans can be medically treated?	1. Yes<br>2. No
AK7	Do you know that there is vaccination for Brucellosis in animals?	1. Yes<br>2. No
AK8	Do you know there is no a vaccination for Brucellosis in humans?	1. Yes<br>2. No
AK9	Do you know that Brucellosis is transmitted by eating /drinking uncooked meat or un-boiled milk?	1. Yes<br>2. No
AK10	Which animals can get infected with Brucellosis?<br>*(Circle all that are applicable if you there are more than one answers)*	1. Cattle<br>2. Goat<br>3. Sheep<br>4. Camel
AK11	Through which method can humans be infected from animals?	1. Eating/drinking raw meat or milk<br>2. Direct contact with brucella contaminated fluids and tissues (*aborted fetus, placenta, secretions /excretion*)<br>3. I do not know

AK12	What are the main symptoms of brucellosis in humans (*mention them)?*	1. Headache<br>2. Prolonged intermittent fever<br>3. Joint, back and /or muscle pain<br>4. Insomnia<br>5. Night sweating and chilling<br>6. Weakness
AK13	What are the main clinical signs /symptoms of brucellosis in animals?<br>*(Circle all that are applicable if you there are more than one answers)*	1. Abortion in the 3rd trimester<br>2. Retained placenta<br>3. Loss of milk production<br>4. Repeat breeding<br>5. Infertility<br>6. Orchitis/epididymitis<br>7. Hygroma
AK14	Was there any awareness raising campaign in your community in the last 12 months about brucellosis?	1. Yes<br>2. No
AK15	If yes to AK14, indicate the date/month/year and by whom	Date ________________________<br>By__________________________
AK16	How do you rate effectiveness of the campaign?	1. Excellent<br>2. Good<br>3. Bad

S2. Risk factor analysis examining the association between *Brucella* sero-positivity and knowledge score (A) and potential risky practices (B) in dairy cattle owners/workers in Maekel and Debub regions, Eritrea.

(A). Mann-Whitney test result: No association was observed between knowledge score and *Brucella* sero-positivity.

Variable	c-ELISA result	Mean	P-value
Knowledge score	Positive	15.33	0.3447
	Negative	6.17	

(B). Chi-square test result: No association was shown between potential risky practices and *Brucella* sero-positivity.

Variable	Category	Number of samples	c-ELISA positive (%)	Chi-square	P- value
Do you drink milk?	Yes	397	5 (1.2)	0.24	0.62
	No	19	0 (0)		
Do you drink raw milk?	Yes	234	2 (0.8)	0.71	0.39
	No	166	3 (1.8)		
Do you use yoghurt from boiled milk?	Yes	30	0	0.41	0.52
	No	370	5 (1.35)		
Do you use yoghurt from raw milk?	Yes	290	4 (1.37)	0.15	0.7
	No	111	1 (0.9)		
Do you eat raw meat?	Yes	31	0	0.41	0.52
	No	385	5 (1.3)		
Do you drink butter milk?	Yes	256	4 (1.5)	0.56	0.45
	No	144	1 (0.7)		
Do you each cheese made from raw milk?	Yes	15	0	0.19	0.65
	No	385	5 (1.3)		
Do you assist animals during parturition?	Yes	312	5 (1.6)	1.67	0.19
	No	104	0		

S3. Brucellosis knowledge of dairy cattle owners/workers in Maekal and Debub regions, Eritrea (n=416). Questions marked with asterisk (*) were only answered by participants who indicated they had heard of brucellosis before (n=210).

Variable	Response	Frequency	Percentage
Have you ever heard about brucellosis before?	Yes	210	50.5
	No	206	49.5
From where did you get the information about brucellosis? *	Television	123	58.6
	Friend/Relative	65	30.9
	Radio	34	16.2
	Community awareness raising program	20	9.5
	Newspaper	3	1.4
Can animals get infected with brucellosis? *	Yes	196	94.2
	I don't know	12	5.8
Which animals can be infected with brucellosis? *	Cattle	192	91.4
	Goats	141	67.1
	Sheep	142	67.6
	Camels	47	22.3
What clinical signs/ are observed in brucellosis diseased animals? *	Abortion	113	53.8
	Repeat breeding	25	12
	Loss of milk production	21	10
	Infertility	10	4.8
	Retained placenta	10	4.8
	Orchitis /epididymitis	4	1.9
	I don't know	86	41
Can brucellosis be transmitted from animals to humans via…? *	Eating/drinking raw meat or milk	178	84.7
	Contact with contaminated fluids and tissues of brucellosis diseased animals	110	52.3
	I don't know	16	7.6
What are the main brucellosis symptoms/clinical sign in humans? *	Joint and back pain	74	35.2
	Fever	46	22
	Weakness	36	17
	Headache	20	10
	Night sweating	9	4.2
	Insomnia	5	2.3
	I don't know	103	49
Can brucellosis in humans be medically treated? *	Yes	181	86.2
	I don't know	19	9
Is vaccination against brucellosis in animals available? *	Yes	146	69.5
	I don't know	52	24.7

S4. Self-reported practices of dairy cattle owners/workers in Maekal and Debub regions, Eritrea (n=416). Questions marked with asterisk (*) were only answered by participants who indicated they assist animals during parturition/calving (n=311).

Variable	Response	Frequency	Percentage
Types and condition of milk and/or milk products consumed	Boiled/ pasteurized	400	96.2
	Yoghurt[1]	290	69.7
	Yoghurt[2]	30	7.3
	Buttermilk	256	61.5
	Cheese	15	3.7
Do you eat raw meat?	Yes	31	7.5
	No	385	92.5
Do you assist animals during parturition/calving?	Yes	311	74.8
	No	103	24.8
Do you wear protective gear (gloves) when assisting calving animals?	Yes	123	39.5
	No	189	60.8

[1]: yoghurt made from raw milk; [2]: yoghurt made from boiled/pasteurized milk

.
